# Evaluation of activation characteristics of a canine platelet concentrate produced by a commercial double centrifugation system

**DOI:** 10.3389/fvets.2024.1384938

**Published:** 2024-05-24

**Authors:** Nicole Tey, Amie Koenig, Katie Hodges, Benjamin M. Brainard

**Affiliations:** Department of Small Animal Medicine and Surgery, College of Veterinary Medicine, University of Georgia, Athens, GA, United States

**Keywords:** veterinary, transfusion medicine, platelet rich plasma, platelet concentrate, thrombocytopenia

## Abstract

**Introduction:**

In veterinary medicine there are few readily available products for platelet transfusion to patients with thrombocytopenia. Commercial tabletop platelet concentrating systems have recently become available to veterinarians, primarily directed towards uses associated with regenerative medicine. These systems could potentially be used to produce fresh concentrated platelets for use in transfusion medicine. This study evaluated the concentration, activation, and sterility of a double centrifugation platelet concentrate (PC) produced by a commercial benchtop system.

**Methods:**

Ten healthy dogs were studied. Whole blood was collected and mixed with ACD-A in a 1:7.6 ratio of ACD-A to whole blood. 12 mL of this mixture was processed into PC via single centrifugation, while 60 mL of the anticoagulated whole blood was processed via a commercial double centrifugation system. Both types of PC were evaluated for platelet concentration, CD62P expression with and without thrombin stimulation, and for sterility.

**Results:**

Mean platelet count in the double centrifuged PC was 863 ± 352 × 10^3^/μL, with very low white blood cell contamination (median of 0.47 × 10^3^ leukocyte/μL (range 0.15–2.18 × 10^3^/μL)). The double-centrifuged PC had similar baseline activation characteristics (as determined by P-selectin expression) as the single centrifuge PC (0.76% vs. 0.72% unstimulated, 30.5% vs. 34.9% stimulated, *p* = 0.432).

**Discussion:**

The benchtop PC system studied here did not cause activation of platelets during production and produced a sterile product that can be further investigated as a source of fresh platelet concentrates for transfusion purposes.

## 1 Introduction

Thrombocytopenia is prevalent among patients admitted to veterinary hospitals. In one retrospective study, 6.7% of hospitalized dogs were thrombocytopenic (1). Severe thrombocytopenia in dogs is considered to be platelet counts below 30,000/μL and may be associated with spontaneous bleeding (2). If hemorrhage occurs in critical areas like the central nervous system or pulmonary parenchyma, it can be difficult to tamponade. Platelet transfusions may promote coagulation in these areas and may be the best way to stop catastrophic hemorrhage due to thrombocytopenia. In other contexts, prophylactic platelet transfusions may be given to patients with severe thrombocytopenia who require invasive procedures, or as a measure until other treatment modalities (e.g., immunosuppression) take effect (3).

In human medicine, clinical practice guidelines recommend prophylactic platelet transfusions for patients with platelet counts of 10,000/μL or less, as they reduce the risk of severe bleeding in this patient population (4). In human patients with chronic thrombocytopenia, prophylactic platelet transfusions decrease the number of bleeding events but have no clear impact on mortality (5). The standard recommended dose in humans is 3 × 10^11^ platelets per person (estimated to be 5 × 10^9^ platelets/kg for an average 60 kg patient), which is about 6 units of pooled platelets or 1 apheresis platelet unit (6).

In veterinary medicine, transfusion products containing platelets are typically limited to fresh whole blood, lyophilized platelets, and leukoreduced cryopreserved canine platelets (7, 8). Fresh platelet concentrates are available for purchase from some commercial blood banks but can be costly and require processing and shipping that may delay vital treatment in patients with life-threatening bleeding. Veterinary facilities with ready access to blood donors can also generate fresh platelet-rich plasma (PRP) or platelet concentrates (PC) through serial centrifugation of whole blood or apheresis. Both techniques can result in high quality platelet products but require dedicated equipment. Fresh platelet products have a short shelf life and are typically generated on an as-needed basis. PRP and PC can be stored at 22–24°C under continuous gentle agitation for up to 5 days. Storage beyond this point may result in decreased platelet hemostatic function and may increase the risk of bacterial contamination (9–11). The use of platelet additive solutions may prolong storage time while limiting storage lesion in concentrated platelet products (9, 12). Refrigerated stored platelets or whole blood may be an option for preservation of functional platelets for longer periods of time, but additional clinical data is needed (7, 8, 13).

Newer benchtop systems that simplify the production of concentrated platelet products have been developed to generate sterile PRP or PC for use in the management of orthopedic and soft tissue injuries. These commercial systems can produce a sterile PC with a high platelet count and low quantities of red and white blood cell contamination with minimal processing by the user (14). Due to the relative ease of production, the PC or PRP produced by these systems may also be a source of platelet products that can be used for transfusion to dogs with thrombocytopenia and make platelet transfusions more readily accessible to veterinary practitioners.

The primary aim of this study was to evaluate the PC produced by a specific commercially available benchtop PC system (Companion Regenerative Therapies Pure PRP, Companion Animal Health) for function and changes occurring during storage, and compare it to PRP produced by a previously reported centrifugation technique (15). Our secondary objective was to evaluate the viability of the PC produced by the commercial system with storage. We hypothesized that the commercial system would create a PC with minimal leukocyte and erythrocyte contamination, and that the activation potential of the platelets as determined by flow cytometry would be comparable to the standard PRP (14). We also hypothesized that the PC would display diminished activation characteristics after 4 days of storage.

## 2 Materials and methods

### 2.1 Sample collection

Ten healthy dogs were recruited for the study. Dogs were included if they were in good general health and not receiving additional medications beyond regular parasiticide treatments. Dogs were required to weigh greater than 20 kg and to tolerate jugular blood sampling without sedation. The recruitment and blood collection protocol were approved by the institution’s clinical research committee (CR-651), and all owners provided signed informed consent.

**Figure 1:**
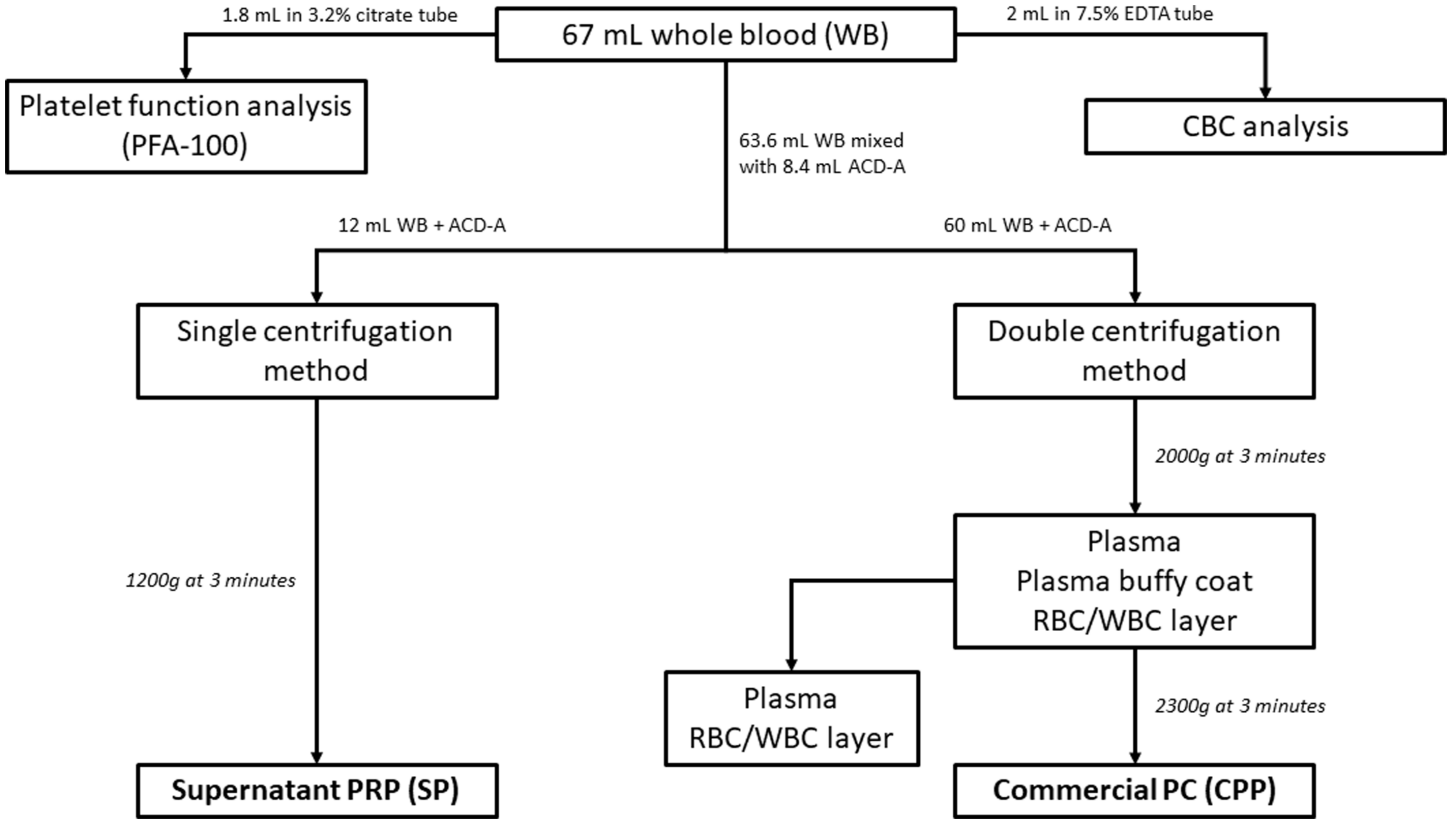
Flowchart of blood collection and processing. All blood was collected via a singular venipuncture using a 19-gauge butterfly catheter and multiple syringes. 1.8 mL of whole blood was added to a 3.2% citrate tube for platelet function analysis and 2 mL of whole blood was added to 7.5% EDTA tube for CBC analysis. The remaining blood was mixed with ACD-A during collection in a 1:7.6 ratio of ACD-A to whole blood. 12 mL of the ACD-A anticoagulated whole blood was processed via single centrifugation to form SP (single centrifugation platelet-rich plasma), while 60 mL of the anticoagulated whole blood was processed via the commercial double centrifugation system to create CPP (commercial platelet product).

For blood collection purposes, the fur over the right or left jugular vein of each donor dog was clipped and the skin was aseptically prepared. Using sterile technique, a 19ga ¾” butterfly catheter (SURFLO Winged Infusion Set, Terumo Corp.) was inserted into a jugular vein and was used to collect all samples (Figure 1): 53 mL of whole blood was collected into a syringe containing 7 mL of acid-citrate-dextrose-A (ACD-A) solution (EmCyte Corporation) to a final volume of 60 mL. An additional 10.6 mL of whole blood was collected into a syringe containing 1.4 mL ACD-A, to a total volume of 12 mL. An additional 3 mL of blood was divided between a tube containing EDTA, which was submitted for a CBC, and one containing 3.2% citrate (for a final 1:9 citrate: blood ratio) for platelet function analysis using a commercial analyzer (PFA-100, Siemens) with a collagen/ADP cartridge (Siemens). Platelet function analysis was performed to ensure appropriate platelet function in all participant dogs.

### 2.2 PRP/PC processing

**Figure 2:**
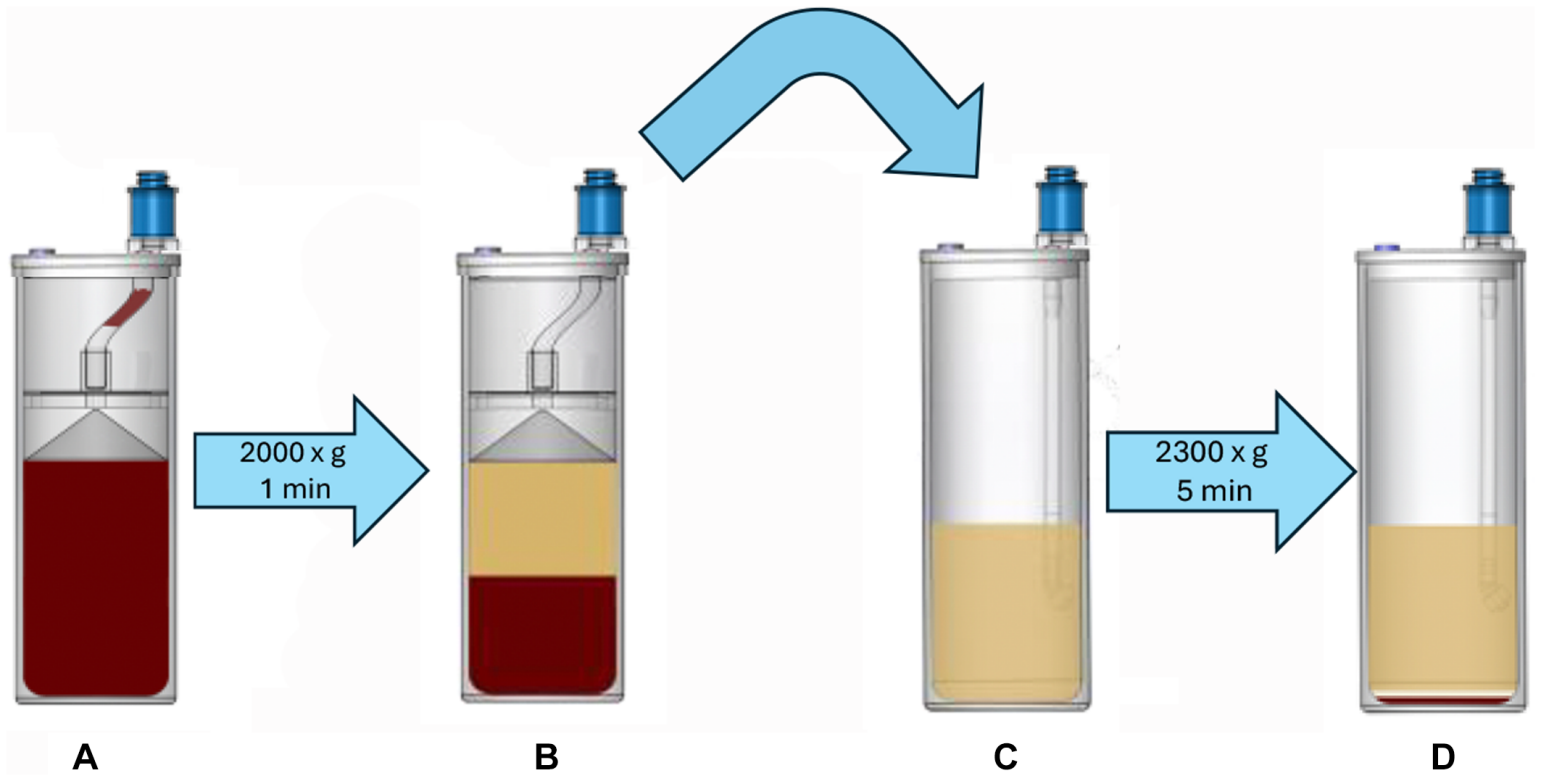
Anticoagulated blood is added to the first concentrating device **(A)**, which is centrifuged at 2,000 × g for 1 min. This process produces a platelet-rich supernatant **(B)**, which is removed and transferred to the second concentrating device **(C)**, which is then centrifuged at 2,300 × g for 5 min, creating a platelet pellet **(D)**. The majority of the platelet-free supernatant is aspirated and discarded, leaving a total of 5 mL of plasma that is used to resuspend the platelet pellet; this forms the platelet concentrate CPP.

Platelet concentrate was prepared from 60 mL of ACD-A anticoagulated blood using a commercially available benchtop system (Companion Regenerative Therapies Pure PRP, Companion Animal Health). The blood was processed according to manufacturer guidelines which includes sequential centrifugation using proprietary concentrating devices (Figure 2). The collected blood was transferred to the first concentrating vial and centrifuged at 2,000 ×g for 1 min to pellet red and white blood cells, leaving a plasma supernatant containing platelets. The supernatant was transferred to a second concentrating device and centrifuged at 2,300 ×g for 5 min to pellet the platelets. All but 5 mL of the platelet-poor supernatant was removed and the remaining platelet poor supernatant was used to resuspend the platelet pellet to create the final product. The output of the commercial system was a standard volume of 5 mL of PC (commercial platelet product, CPP). After processing and analysis on day 0, the CPP samples were stored at room temperature (23–24°C) in capped plastic syringes on a flat oscillating platform providing constant gentle agitation.

The remaining 12 mL of ACD-A-anticoagulated blood was transferred from the syringe into a 15 mL conical tube and centrifuged at 1,200 ×g for 3 min at 24°C with no brake applied (16). A variable amount of supernatant PRP (SP) was produced and collected for study as a control sample for flow cytometric analyses.

### 2.3 CBC and blood gas analysis

On day 0, both CPP and SP had a full CBC performed (Advia 2120, Siemens Healthcare). CPP samples had platelet and cell counts repeated on Days 1 and 4 using the same analyzer, which were compared to the initial CBC results. Blood gas analysis (NOVA PhOX ultra, NOVA Biomedical) was performed on CPP samples on day 0 and day 4. These were compared to the baseline CBC performed on the EDTA-anticoagulated whole blood from the initial collection.

### 2.4 Sterility

The CPP samples underwent aerobic bacterial culture on Day 0 and Day 4. Sterile disposable spreaders inoculated 100 μL of the CPP sample onto blood agar/MacConkey biplates (Blood Agar 5%/MacConkey Agar biplate, Hardy Diagnostics). The plates were incubated at 37° C for 72 h and visually assessed for bacterial growth. Any bacterial colonies were collected and submitted to a commercial microbiology laboratory for identification.

### 2.5 Flow cytometry

Both CPP and SP samples were analyzed for responsiveness to thrombin on Day 0. CPP samples were analyzed again on Day 4, using techniques that have been described elsewhere (17). Briefly, samples of each platelet product (CPP, SP) were diluted with wash buffer (137 mM NaCl, 4 mM KCl, 0.5 mM Na_2_HPO_4_, 0.5 mM MgCl-6H_2_O, 0.1% glucose, 0.2% bovine serum albumin, 10 mM HEPES) to a platelet count of 5,000/μL. Gly-Pro-Arg-Pro amide (20 mM; Sigma Aldrich) was added to each reaction to inhibit fibrin polymerization. Bovine thrombin (final concentration of 1 U/mL, Sigma Aldrich) diluted in HEPES-buffered saline (10 mM HEPES, 150 mM NaCl) or an equal volume of HEPES-buffered saline was added to the platelet solutions and incubated for 15 min at room temperature (23°C).

Following this incubation, fluorescence-conjugated antibodies against CD61 (Clone F11, Bio-Rad) and CD62P (Clone A1.2, BD Biosciences) were added to the reaction mixtures (17). Mouse IgG1-PE isotype control (Clone MOPC-2, BD Pharmingen) was included as a negative control for CD62P in two separate samples (with and without thrombin). Following a 20 min incubation in the dark at room temperature, the reactions were diluted with 250 μL of platelet wash buffer and analyzed by flow cytometry (NovoCyte Quanteon 4025 flow cytometer with NovoSampler Q, Agilent Technologies) with data collected using proprietary software (NovoExpress version 1.5.6 software, Agilent Technologies). A digital compensation matrix including all fluorochromes was established and applied during data acquisition to compensate for spectral overlap.

**Figure 3:**
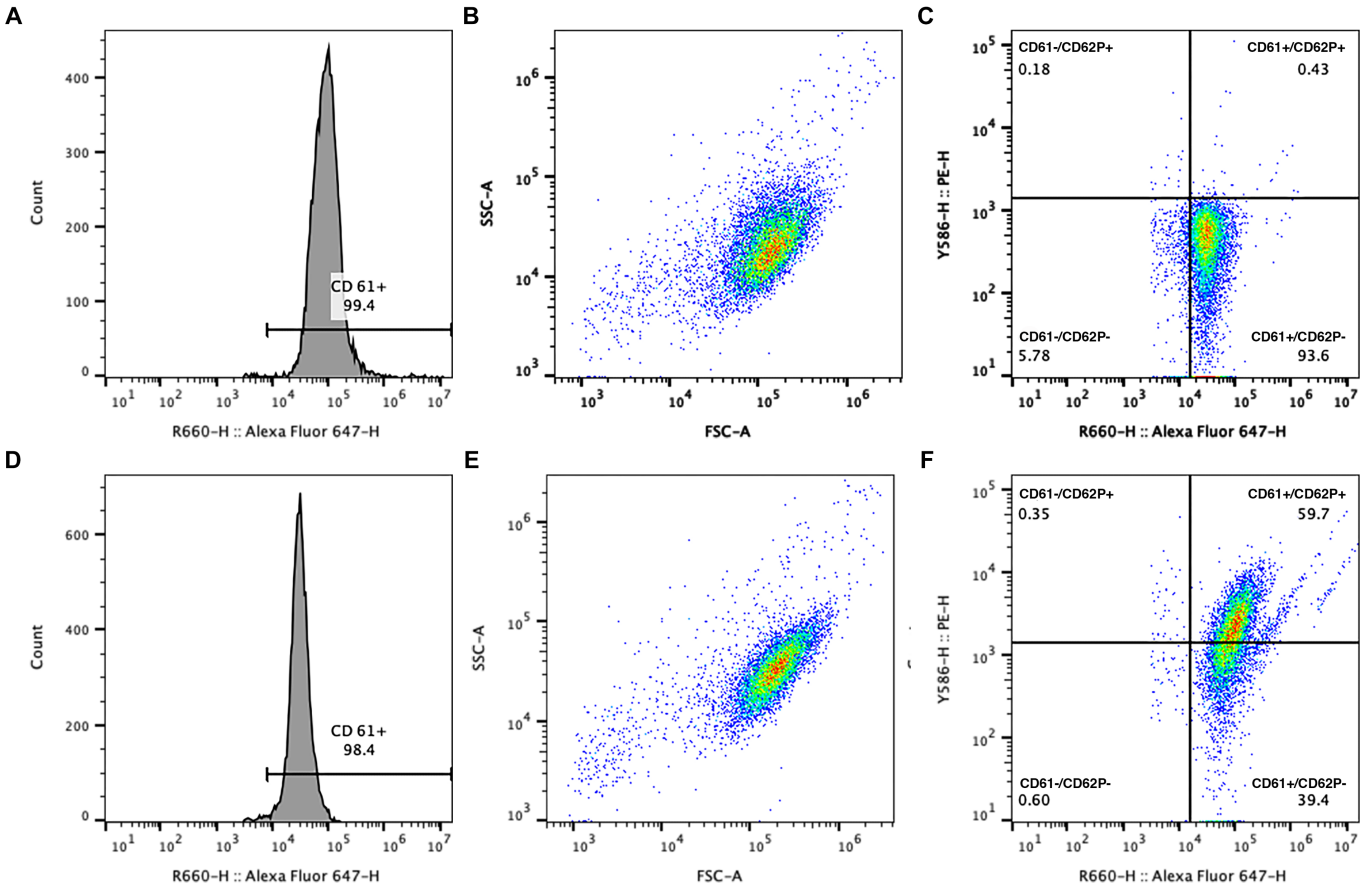
Example gating logic for analysis of CD62P expression in platelet concentrates with **(D–F)** and without **(A–C)** the addition of 1 U/mL thrombin. Platelets were identified using CD61 positivity **(A,D)** and a characteristic forward and side scatter histogram **(B,E)**. CD62P-PE was used to evaluate P-selectin expression on platelets, indicated by events in the upper right quadrant of the dual fluorescence graph (**C,F**, with CD61-AF647 expression on the X axis and CD62P-PE expression on the Y axis). Response to thrombin was appreciated as an increase in the percentage of CD61+ platelets that showed CD62P expression **(F)**.

**Figure 4:**
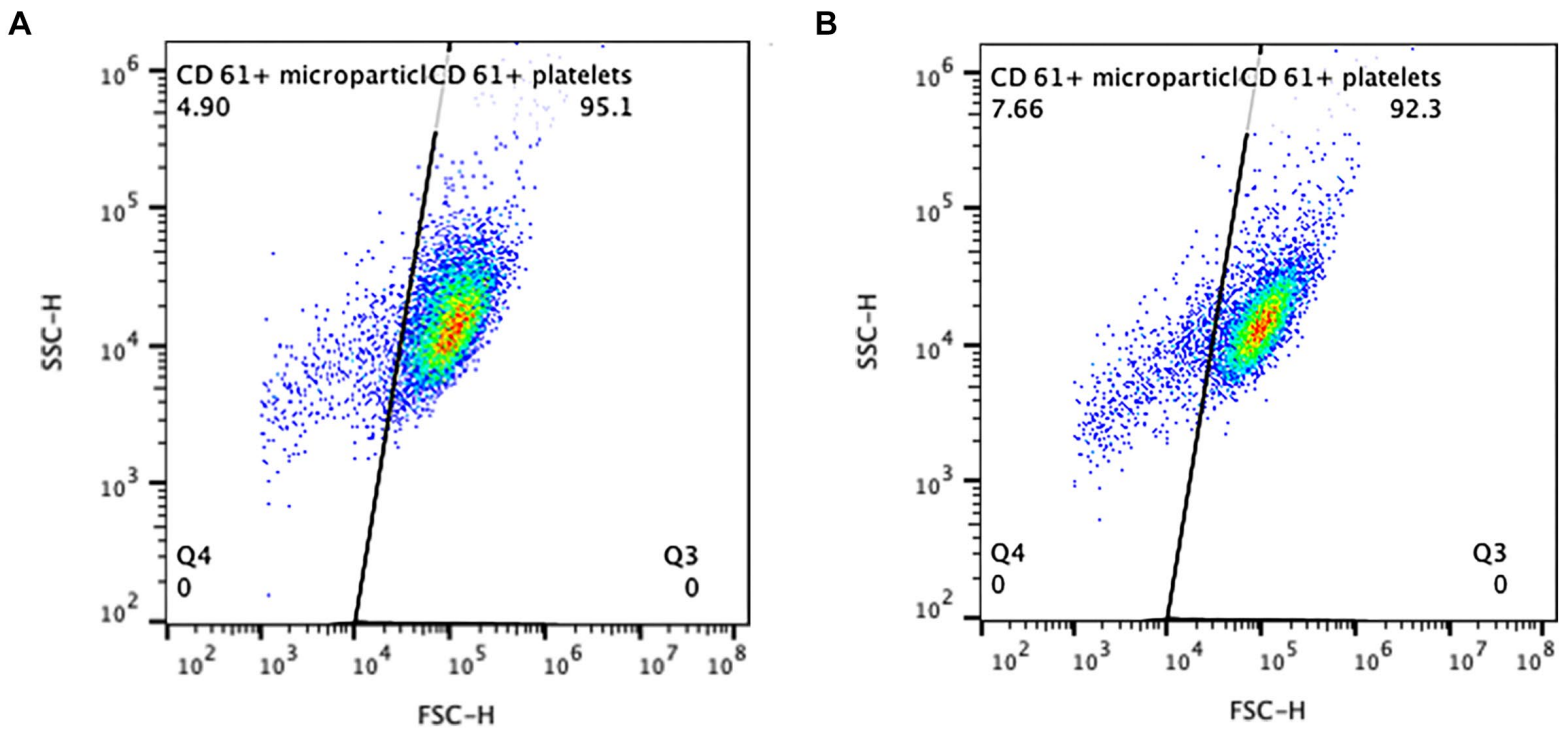
Presumptive microparticles were identified as the population of CD61+ events smaller in size (to the left) of the main platelet cloud, distinguished by the diagonal gate. The CD61+ events in this gate were considered to be particles of platelet origin. Two examples of the CPP samples from a single dog on day 0 (**A**, 4.9% microparticles) and day 4 (**B**, 7.66% microparticles), are shown.

Flow cytometric data was collected to include 10,000 putative single platelets per run. Platelets were identified through CD61 expression and characteristic forward (diffracted) and side (refracted) scatter (Figure 3). Expression of CD62P in platelets with and without activation by thrombin was recorded as percentage of total particles in the quadrant representing the population of CD61+ and CD62P+ events. A threshold of positivity for CD62P expression was defined based on the fluorescence of the isotype-PE-labeled platelets and was confirmed as biologically relevant when applied to the sample data from the unactivated platelets. The percentage of CD61+ particles smaller than the identified mature platelets was evaluated as platelet microparticles (Figure 4). Flow cytometric data was analyzed using commercial flow cytometric software (FlowJo software v.10.3, BD Biosciences).

### 2.6 Statistical analysis

Statistical analysis was performed using commercially available statistical software (Prism v. 8.4.3, GraphPad software; Sigmaplot v. 14, Systat software, Inc.; SAS v. 9.4, SAS Institute). Data distribution was assessed for normality using a Shapiro–Wilk test. Normally distributed data are summarized as mean ± standard deviation, and non-parametric data are described as median (minimum-maximum values). Comparison between CPP and SP was performed using paired t-tests or Wilcoxon rank sum analysis, depending on the source and distribution of the data. In the CPP data, comparisons across days were accomplished using a one-way ANOVA for repeated measures, or a Friedman test for non-parametric data. When indicated, corrections for multiple comparisons were performed using Dunn’s multiple comparison test. Evaluation of the differences in CD61 and CD62P expression between CPP and SP with and without thrombin activation on day 0 was done with a linear mixed model. Comparisons of values post activation to pre-activation for each method and day were made. *p*-values were adjusted for multiplicity using the false discovery methods of Benjamini and Hochberg for each variable separately. The interactions of method and activation and of activation and day were tested to compare the change in variables due to activation between methods and days. A significance threshold of *p* < 0.05 was used for all statistical analyses.

## 3 Results

The mean age of the dogs was 4.7 ± 2.8 y (range 2–9 y), and mean weight was 31 ± 5.5 kg, (range 23.4–41 kg). The mean baseline platelet count from the CBC for the enrolled dogs was 214 ± 70 × 10^3^/μL (reference interval 150 × 10^3^–500 × 10^3^/μL). One dog had a CBC platelet count of 53 × 10^3^/μL but was included in the study because the post-centrifugation samples concentrated to a platelet count that was similar to the other samples (suspecting an unobserved clot in the tube used for CBC analysis). The PFA-100 results for all dogs had a mean closure time of 75.75 ± 19.8 s (reference interval 56–85 s). Baseline hematocrit for all dogs was 44.4 ± 3.9% (median 43.7%, range 37.5–50.1%), and all were within the institutional reference interval (37.5–55.6%).

### 3.1 Platelet concentration

Following processing, CPP samples had a mean platelet count of 863 ± 352 × 10^3^/μL (range 218–1,285 × 10^3^/μL), while SP samples had a mean platelet count of 213 ± 95 × 10^3^/μL (range 70–351 × 10^3^/μL, *p* = 0.0005). CPP samples produced a standardized volume of 5 mL of platelet product, while SP samples had volumes that were variable between 2 and 5 mL. CPP had a median of 4.3 × 10^9^ platelets/sample (range 1.09 × 10^9^–6.4 × 10^9^ platelets/sample). The CPP platelet counts were significantly different from the baseline (whole blood) platelet count (*p* = 0.0007), while the SP platelets were not (*p* = 0.995).

On visual inspection, all CPP samples had a slight pink tint, but the hematocrit was assessed as 0% for all samples in both CPP and SP groups. All dogs had median baseline leukocyte counts of 7.17 × 10^3^/μL (range 4.41–9.28 × 10^3^/μL; reference interval: 4.2–13.2 × 10^3^/μL). The CPP samples had a median leukocyte count of 0.47 × 10^3^/μL (range 0.15–2.18 × 10^3^/μL), while SP samples had a median leukocyte count of 0.05 × 10^3^/μL (range 0.01–0.44 × 10^3^/μL). Both the CPP and SP leukocyte counts were decreased from baseline (*p* < 0.0001).

On Day 1, the platelet count of CPP samples had not decreased significantly from the day 0 platelet count (795.7 ± 353.0 × 10^3^/μL; *p* = 0.335). On Day 4, the mean platelet count had decreased to 673.5 ± 317.9 × 10^3^/μL (*p* = 0.009 compared to day 1 and *p* = 0.004 compared to day 0).

### 3.2 Blood gas analysis

The mean pH in the CPP samples following processing on day 0 was 7.405 ± 0.11, with a mean PCO_2_ of 17.96 ± 3.84 mmHg and a mean lactate concentration of 0.71 ± 0.3 mmol/L. On day 4, a repeat analysis of the CPP samples failed to register a pH in all but 2 samples (indicating a value lower than 6.500). The mean pH in 2 samples that gave a measurable value was 6.985 ± 0.127. The mean PCO_2_ of the day 4 CPP samples was 88.85 ± 45.11 mmHg (10 out of 11 samples outside reference intervals for venous PCO_2_), greater than that of day 0 (*p* = 0.001). Only 3 samples had a measurable lactate concentration on day 4, with a mean of 11.53 ± 6.9 mmol/L. The 7 samples with an unmeasurable lactate concentration all had concentrations above the analyzer limit of quantification (20 mmol/L).

### 3.3 Platelet activation

**Figure 5:**
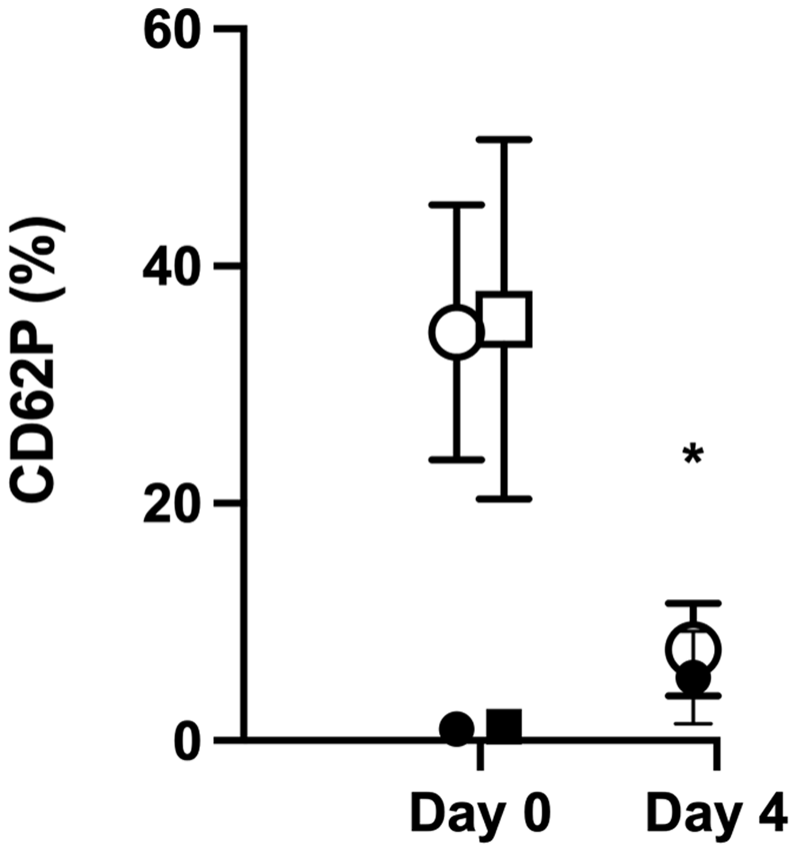
CD62P expression was similar between CPP and SP on day 0 without stimulation and with stimulation. On day 4, flow cytometry was repeated on CPP samples, and there was a significantly increased baseline expression of CD62P without thrombin stimulation (indicated by a *; *p* = 0.004), but the addition of thrombin did not result in a significant increase in CD62P expression (*p* = 0.083). The degree of CD62P expression following thrombin stimulation was significantly lower than the expression in thrombin-activated samples on day 0 (*p* < 0.001).

Day 0 CD62P expression in unstimulated platelets was not different between CPP and SP (medians of 0.76% [0.22–3.1%] and 0.72% [0.36–4.7%], respectively, *p* = 0.432). Following stimulation with thrombin, P-selectin expression was increased from baseline (*p* < 0.001) in both groups (to medians of 30.5% [24.6–58%] and 34.9% [14–59.7%], respectively), but the degree of CD62P expression was not different between CP and SP samples (*p* = 0.652). On day 4, the CPP group prior to activation with thrombin had a significantly increased CD62P expression compared to day 0 (median of 6.03% [0.69–13.01]; *p* = 0.004) but following addition of thrombin did not increase P-selectin expression (median of 7.46% [1.11–16.20%], *p* = 0.083). This degree of CD62P expression was significantly lower than the expression in thrombin-activated samples on day 0 (*p* < 0.001) (Figure 5).

The percentage of CD61+ microparticles on day 0 in the unstimulated CPP and SP samples was slightly higher in the SP sample (means of 4.0 ± 1.9% and 4.7 ± 2.2%, respectively, *p* = 0.004). Following thrombin stimulation, the means were not significantly different from the unstimulated samples in the CPP group (mean of 5.5 ± 2.6%, *p* = 0.125) nor the SP group (6.7 ± 3.9%, *p* = 0.063). The post-stimulation CPP and SP samples were not different from each other (*p* = 0.079). On day 4 in the CPP group, the mean percent CD61 expression in the unstimulated microparticles was 8.3 ± 6.7%, and that in the stimulated samples was 8.2 ± 6.2% (*p* = 0.700). There was no difference in the percent CD61 expression in the microparticle gate on day 4 compared to day 0 in either the unstimulated or stimulated samples (*p* = 0.083 and *p* = 0.247, respectively).

### 3.4 Bacterial cultures

One plate grew two small colonies on Day 4. These were identified as *Neisseria mucosa/sicca* and *Streptococcus mitis*. The Day 0 plate from the same donor was negative for growth.

## 4 Discussion

The commercial benchtop PRP system described here generated a PC with minimal leukocyte and erythrocyte contamination. Based on flow cytometric analysis, these platelets showed a similar response to thrombin stimulation as PRP produced by a single centrifugation (thought to be less traumatic to the platelets than the double centrifugation method). In addition, the PC appeared to be sterile, based on aerobic bacterial culture, after initial preparation and after storage at room temperature for 4 days. The PC, however, did not maintain responsiveness over the 4-day storage period. Flow cytometric analysis on day 4 revealed a significant decrease in responsiveness to thrombin stimulation. The samples also had deranged blood gas results, with high lactate levels and low pH indicative of platelet storage lesions (18, 19). Despite the other changes indicative of platelet storage lesion, there were no changes in the amount of CD61+ microparticles, which were expected to increase with ongoing platelet activation and cell death.

The observed decrease in platelet responsiveness may have been a result of the storage protocol rather than the product itself. Standard blood and platelet collection bags are made of polyvinylchloride (PVC), and the thickness of this material can influence gas exchange during storage (20). A recent report on a leukoreduced PC that was produced through sequential centrifugation used a PVC storage bag designed specifically for PC. Based on the blood gas results for the stored PC in this report, this bag allows for transfer of both CO_2_ and oxygen away from and into the stored platelets (21, 22). Storage of the CPP in capped polypropylene syringes in the current study likely impaired any gas exchange with the environment. We were unable to locate suitably sized (5 mL) PVC storage bags for use in this study, however the transfer of the PC in this study to an appropriate storage bag following preparation may have improved overall health during storage.

The addition of platelet additive solutions (PAS) could also have prolonged shelf life and improved PC health, with decreased development of platelet storage lesions (9, 23). A recent report evaluated Plasma-Lyte A solution as an additive solution for canine PC that was produced through sequential centrifugation (9). This study concluded that the use of this PAS, in combination with cold storage over 21 days, resulted in minimal evidence of platelet storage lesion with maintained platelet function. The PC collected in the current study were stored on a flat orbital shaker to provide gentle continuous agitation, which is associated with better platelet viability in human studies, but which could not overcome the impact of storage in a syringe and without PAS (20).

Although one culture plate did show growth of two single colonies of bacteria, these most likely represent contamination during the plating process rather than of the sample, due to the minimal amount of bacterial growth (a single colony of each) and the specific species that were grown. If methodology can be devised that allows prolonged storage of PC, a more in-depth assessment of sterility of the product is recommended prior to recommendations for indications that demand sterility. There has also been some interest in human and veterinary medicine in the refrigerated storage of whole blood as well as platelet concentrates, which may help to deter bacterial growth and allow prolonged storage (13, 24). Refrigerated storage may also decrease the metabolic rate of the platelets and delay generation of storage lesions. In addition, cold-stored platelets may have augmented hemostatic capabilities and so may be of additional benefit in patients with thrombocytopenic hemorrhage (25).

The lack of a centralized blood bank system in veterinary medicine limits the availability of platelets for transfusion to treat patients hemorrhage caused by severe thrombocytopenia. Without the infrastructure to process whole blood donations into component therapy such as platelet concentrates or PRP, many veterinary practices are constrained to the administration of whole blood transfusions, cryopreserved platelets, or lyophilized platelets. Whole blood transfusions come with increased risk of circulatory overload due to the volume of blood product administered. Other transfusion reactions can also occur from exposure to antigens on the erythrocytes, immune cells, or in the plasma of the donor dog. Platelet apheresis can be performed to create a platelet concentrate and is generally well tolerated but requires expensive equipment and can expose donors to complications such as hypocalcemia, hypomagnesemia, and hypotension (26). The utility in commercial PRP systems is that they can serve dual functions in orthopedic or rehabilitative medicine as well as in transfusion medicine and may already be present in many clinics for the former application. The systems also have a small footprint (especially compared to a conventional blood centrifuge) and can be readily integrated into a hospital even without a dedicated blood bank service. The use of pre-made PRP kits and reagents systematizes the PC creation process, limiting inter-user variability.

The median number of platelets acquired from CPP samples in this study was 4.3 × 10^9^ platelets. The standard dose of platelets typically recommended for infusion to canine patients with thrombocytopenia is approximately 8 × 10^10^ platelets/10 kg (27). This dosing convention is based on traditionally recommended doses in human medicine (4). There is a paucity of data on the appropriate platelet doses for animals with hemorrhage, which likely varies with the cause of thrombocytopenia. More specifically, the dose of platelets necessary to stop bleeding in a thrombocytopenic animal may not be the same dose that would be required to increase circulating platelet count (which is the basis for the human recommendations). If a lower transfused number of platelets is adequate to stop bleeding, a higher targeted transfusion volume may not be necessary. The most common cause for severe thrombocytopenia in our canine patient population is immune-mediated thrombocytopenia and smaller breeds like Cocker Spaniels, Miniature and Toy Poodles, Havanese, and Maltese are overrepresented in this group; thus, a lower total number of platelets may be adequate to achieve the desired hemostatic effect (1, 28). Another consideration is that the volume of blood obtained to create this dose of CPP platelets was about 53 mL of whole blood, which is 1/9–1/10th of a standard blood donation. If a larger dose of platelets is desired, more PC can be generated from the same donor by collecting more blood.

Limitations of this study include the small sample size and lack of additional avenues of function testing of the products. Further comparative testing, such as platelet aggregometry, would have been an additional method to assess the *in vitro* function of the PC and the response to other agonists besides thrombin. The standard centrifugation technique failed to concentrate platelets as previously documented (16), but we do not feel that this invalidated the use of the SP platelets as comparator for the PC group for flow cytometric assessment. Flow cytometry requires only a small number of platelets for analysis and was unlikely to have been impacted by the concentration of either sample. There are multiple methods with which PRP can be generated, and other centrifuge settings may have resulted in better concentrated PRP; or a double-centrifuge method could have been used to make the SP concentration and volume more comparable (29). In addition, the methodology for determination of platelet microparticles was based solely on the flow cytometric appearance of the main platelet (CD61+) population. As such, smaller platelets, as well as platelet fragments may have been inadvertently counted as microparticles. In addition, the possibility that changes in light scatter following activation resulted in an apparently smaller sized platelet cannot be ruled out.

In conclusion, assays performed on platelet concentrates created by the commercial PRP system indicate the samples are well-concentrated, sterile, have minimal leukocyte and erythrocyte contamination and have similar thrombin response to fresh platelet-rich plasma created via standard centrifugation at the time of collection. Additional *in vivo* studies to assess hemostatic function of CPP are warranted.

## Data availability statement

The raw data supporting the conclusions of this article will be made available by the authors, without undue reservation.

## Ethics statement

The animal studies were approved by the University of Georgia Clinical Research Committee. The studies were conducted in accordance with the local legislation and institutional requirements. Written informed consent was obtained from the owners for the participation of their animals in this study.

## Author contributions

NT: Formal analysis, Investigation, Writing – original draft, Writing – review & editing. AK: Writing – review & editing. KH: Investigation, Writing – original draft. BB: Conceptualization, Data curation, Formal analysis, Funding acquisition, Investigation, Methodology, Supervision, Writing – original draft, Writing – review & editing.
